# Long-Term Results of Isolated Latissimus Dorsi to Rotator Cuff Transfer in Brachial Plexus Birth Injury

**DOI:** 10.1055/s-0044-1786817

**Published:** 2024-06-12

**Authors:** David J. Kirby, Daniel B. Buchalter, Lauren Santiesteban, Mekka R. Garcia, Aaron Berger, Jacques Hacquebord, John A.I. Grossman, Andrew E. Price

**Affiliations:** 1 Department of Orthopedic Surgery, NYU Langone Health, New York, New York, United States; 2 Department of Neurology, NYU Langone Health, New York, New York, United States; 3 Deparment of Orthopedic Surgery, Nicklaus Children's Hospital, Miami, Florida, United States

**Keywords:** brachial plexus birth injury, latissimus transfer, brachial plexus palsy, global palsy, upper trunk palsy, clarion sign

## Abstract

**Background** Brachial plexus birth injury results in deficits in strength and motion, occasionally requiring surgery to restore power to the deficient external rotators of the shoulder in these patients. This is a retrospective analysis of the long-term results of an isolated latissimus dorsi transfer to the rotator cuff in patients with brachial plexus birth injury.

**Methods** This is a retrospective review of prospectively collected data for patients undergoing isolated latissimus dorsi transfer into the infraspinatus in addition to release of the internal rotation contracture of the shoulder with greater than 5 years' follow-up. Preoperative and postoperative shoulder elevation and external rotation were documented. Failure of surgery was defined as a return of the internal rotation contracture and a clinically apparent clarion sign.

**Results** A total of 22 patients satisfied the inclusion criteria: 9 global palsies and 13 upper trunk palsies. The average follow-up was 11 years, ranging from 7.5 to 15.9 years. There was a trend for improved external rotation in the global palsy cohort at final follow-up ( *p* = 0.084). All nine global palsies maintained adequate external rotation without a clarion sign. Five of the 13 upper trunk palsies failed the latissimus dorsi transfer and subsequently required either teres major transfer and/or rotational osteotomy. In these five failures, the period from initial transfer to failure averaged 6.6 years, ranging from 3.4 to 9.5 years.

**Conclusion** The results of this study indicate that patients with global palsy have sustained long-term improved outcomes with isolated latissimus dorsi transfer while patients with upper trunk palsy have a high rate of failure. Based on these results, we recommend isolated latissimus dorsi transfer for global palsy patients who have isolated infraspinatus weakness.

**Level of Evidence:** Case series – Level IV.

## Introduction

Brachial plexus birth injury (BPBI) can result in deficits in shoulder function including strength and motion. Epidemiologic studies have cited the incidence of BPBI as 0.4 to 4 per 1,000 births. 1 2 3 The most common injury to the brachial plexus involves the C5-C6 ± C7 nerve roots, that is, upper trunk palsy; the next most common injury is the global palsy, which involves all the nerve roots (C5-C8, T1). Although the vast majority of patients recover spontaneously from BPBI, persistent deficits can lead to severe limitations with internal rotation contracture and difficulty in forward elevation of shoulder function. Initial explanations for the internal rotation contracture of the shoulder were attributed to unopposed adductors and internal rotators, which are either unaffected or less affected by the injury (pectoralis major, coracobrachialis, pectoralis minor, subscapularis, teres major, and latissimus dorsi) compared with the weak external rotators and abductors (supraspinatus, infraspinatus, posterior, and middle fibers of the deltoid). 4 5 However, recent studies provide evidence that the contracture is due to impaired longitudinal muscle growth as a result of denervation relative to skeletal growth. 6

BPBI can range in severity from minor traction injuries to complete root avulsion injuries. Treatment begins with physical and/or occupational therapy to assist in maintenance of flexibility and range of motion. Early surgical intervention with exploration of the plexus and grafting is indicated in those who require reinnervation of dysfunctional muscles. Despite early intervention, patients may experience incomplete neurologic recovery and can require secondary surgical procedures, especially for those with residual internal rotation contractures. The most widely utilized procedures are those modified from various historical operations.

Fairbank first noted improvement in external rotation with release of the subscapularis muscle and the joint capsule. 7 Sever then performed release of both the subscapularis and the pectoralis major tendons with osteotomy of the coracoid. 8 L'Episcopo added to this operation by transferring the latissimus dorsi and teres major to the lateral humerus under an osteoperiosteal flap to provide active motor function to a released shoulder. 9 Hoffer later transferred the latissimus dorsi and teres major tendons into the rotator cuff. 10 11 Ultimately, release of the contracture and muscle transfer of the teres major and/or latissimus dorsi have been used to restore power to the deficient external rotators of the shoulder in these patients. 12

Untreated BPBI leads to progressive glenohumeral joint deformity with glenoid dysplasia, retroversion, and flattening of the humeral head, which subsequently leads to posterior displacement of the humeral head. 13 14 15 However, there is no current consensus on the ideal treatment, which can range from isolated arthroscopic anterior capsule release to open teres major and latissimus dorsi transfer. Additionally, optimal management of upper trunk palsy versus global palsy has not been thoroughly parsed out. The aim of this study was to evaluate the long-term outcomes of isolated latissimus dorsi transfer in patients with upper trunk and global BPBI. We hypothesize that that the latissimus dorsi transfer with contracture release would have improved shoulder range of motion in both global palsy and upper trunk palsy at 5+ years' follow-up.

## Materials and Methods

This was a retrospective study of patients undergoing isolated latissimus dorsi transfers by a single surgeon who performed all procedures. To be eligible for the study, the following inclusion criteria were applied: (1) a diagnosis of BPBI with a functionally disturbing internal rotation contracture with clarion sign; (2) a congruent glenohumeral joint documented on computed tomography or magnetic resonance imaging scan; (3) greater than 1 year of age at surgery; (4) undergoing isolated latissimus dorsi transfer into the infraspinatus in conjunction with release of the internal rotation contracture; and (5) a minimum of 5 years of follow-up. No preoperative electromyography was required for diagnosis. Surgical indication was a history of BPBI with persistent internal rotation contracture, infraspinatus power of less than ⅖ (active movement only with gravity eliminated), and teres minor power greater than or equal to ⅘ (active movement against gravity with some resistance), using the Medical Research Council scale.

Preoperatively, 52 patients were identified who underwent an isolated latissimus dorsi transfer for an internal rotation contracture, 22 of whom had a minimum of 5 years' follow-up. Physical examination was performed by the senior author preoperatively and at postoperative follow-up documenting range of motion for shoulder elevation and external rotation, as well as evaluation for clarion or trumpet sign (almost 90 degrees abduction of affected arm when bringing hand to the mouth). Demographics and patient-specific characteristics were prospectively collected. Patients were divided into two groups based upon the extent of neural involvement: upper trunk palsy (C5-C6 ± C7 involvement) or global palsy (C5-C8, T1 involvement). Patients were closely followed clinically postoperatively. Failure was defined as a return of the functionally limiting internal rotation contracture, in addition to a clinically apparent clarion sign.

### Operative Technique

**Fig. 1:**
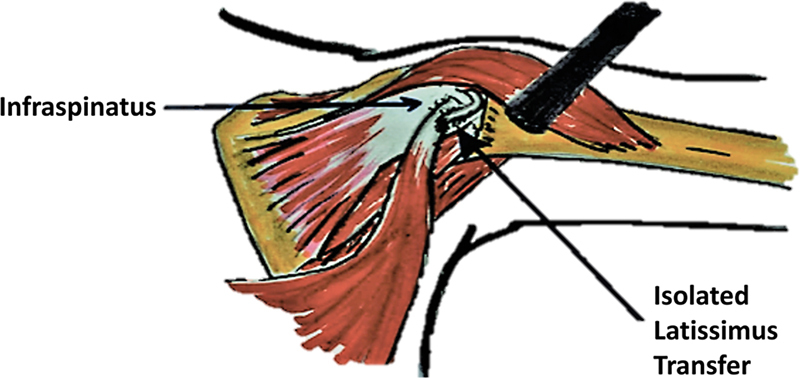
Operative technique.

The patient is placed in the supine position with a small bump placed under the scapula. The internal rotation contracture release and latissimus dorsi transfer are performed through a longitudinal midaxillary incision that proximally is curved posteriorly. A subscapularis slide is performed as previously described. 16 The latissimus dorsi muscle/tendon is then separated from the teres major and released off of its insertion on the humerus. Care is taken to identify and protect the neurovascular pedicle to the latissimus dorsi. The latissimus dorsi tendon is brought over the long head of the triceps, woven through the infraspinatus tendon at its insertion on the humerus, and then sewn onto itself using nonabsorbable suture ( Fig. 1 ). The wound is irrigated extensively, and hemostasis is achieved via electrocautery. The incision is closed via a layered technique using subcutaneous, absorbable sutures.

### Postoperative Care

Postoperatively, all patients were placed into a shoulder spica cast with the shoulder positioned in 60 degrees of external rotation and 30 degrees of abduction for 6 weeks. Occupational therapy started after cast removal.

### Statistical Analysis

Statistical evaluation was performed with Excel version 16.0 (Microsoft Corporation, Redmond, Washington, United States) and GraphPad Prism software version 8.3.0 (GraphPad Software, La Jolla, California, United States). Numerical demographic data (age and follow-up) was compared using an unpaired ordinary one-way analysis of variance. Categorical demographic data (gender and laterality) was analyzed using a Fisher's exact test. Preoperative and postoperative range of motion data was analyzed using unpaired parametric Student's *t* -tests. Surgical success was statistically analyzed using Kaplan–Meier estimation with log-rank (Mantel–Cox) test. In all cases, *p* < 0.05 was considered significant.

## Results

There were 52 patients who underwent isolated latissimus dorsi transfers performed for internal rotation contractures in BPBI patients from 2000 to 2010. After applying exclusion criteria, 22 patients were identified with at least 5 years of follow-up. Of these, there were 13 upper trunk palsy and 9 global palsy patients ( Table 1 ). Thirteen patients were female (9 upper trunk, 4 global palsies) and 9 patients were male (4 upper trunk and 5 global palsies). The average length of follow-up was 11.2 years, ranging from 7.5 to 15.9 years. No statistical difference was noted between palsy types regarding age, gender, laterality, and follow-up ( Table 1 ).

**Table 1:** Patient demographics

Variable	Total cohort ( *N* = 22)	Global palsy cohort ( *N* = 9)	Upper trunk palsy cohort ( *N* = 13)	*p* -Value (global vs. upper trunk)
**Age (y)**	4.49 ± 1.9	4.69 ± 2.4	4.35 ± 1.5	> 0.99
**Gender (female)**	40.9	55.6	30.8	0.38
**Palsy laterality (right)**	36.4	33.3	38.46	> 0.99
**Follow-up time (y)**	11.2 ± 2.5	11.2 ± 3.1	11.1 ± 2.1	> 0.99

Note: Data are shown as mean ± standard deviation and number of patients and hips (%).

**Fig. 2:**
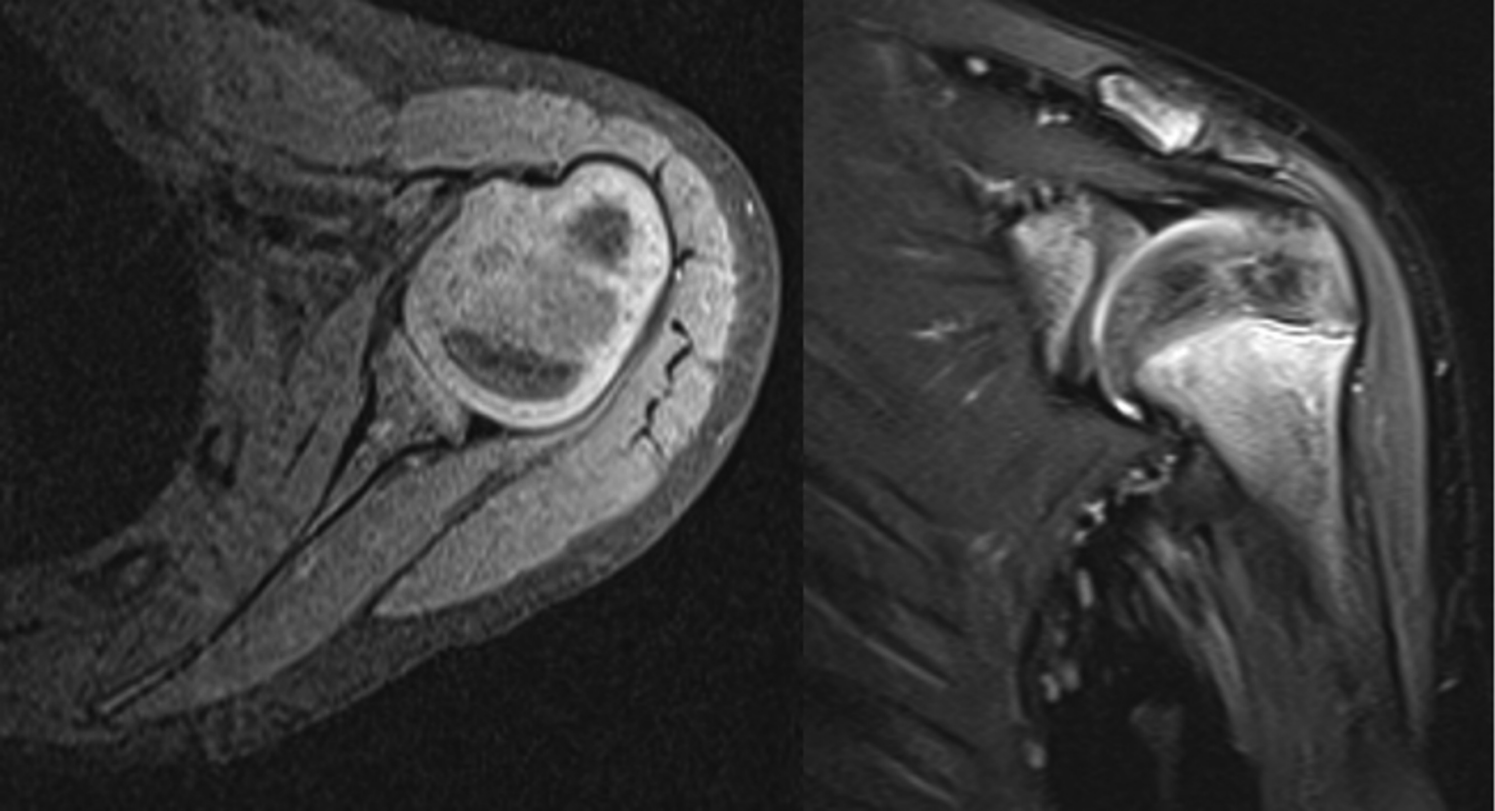
Representative magnetic resonance imaging (MRI) of glenohumeral joint in patient with global brachial plexus injury.

**Fig. 3:**
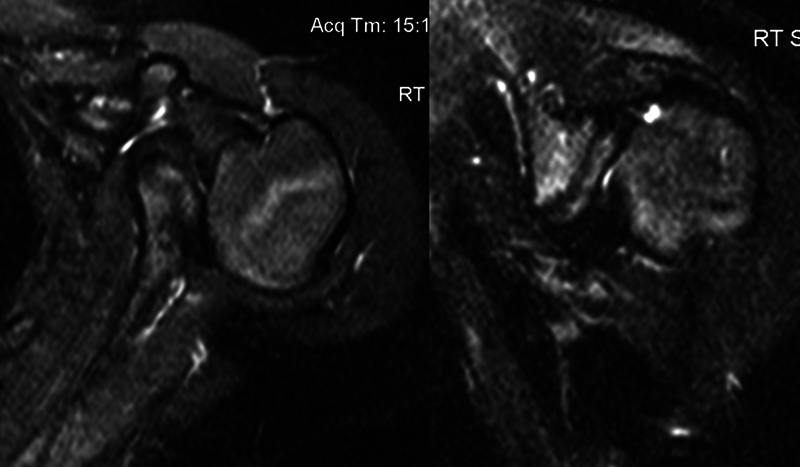
Representative magnetic resonance imaging MRI) of glenohumeral joint in patient with upper trunk brachial plexus injury.

Preoperative and postoperative active range of motion results are summarized in Table 2 . The average preoperative elevation for all patients was 115.5 degrees (ranging from 45 to 170 degrees) which improved to 126.6 degrees (ranging from 30 to 170 degrees) at final follow-up; the average preoperative external rotation for all patients was 24.8 degrees (ranging from –10 to 75 degrees) which improved to 36.7 degrees (ranging from –5 to 80 degrees) at final follow-up. There was no statistically significant difference in preoperative to postoperative elevation or external rotation for the total cohort. There was a strong trend for improved external rotation in the global palsy cohort, which improved from 32.5 to 55.0 degrees ( *p* = 0.084). The upper trunk palsy cohort had greater elevation preoperatively and at postoperative follow-up compared with the global palsy cohort, while the global palsy cohort had a greater improvement in external rotation at follow-up compared with the upper trunk palsy cohort, all of which were statistically significant. For patients with upper trunk palsy, those that had a successful surgical outcome had significantly better external rotation than those that failed ( *p* = 0.0002). On follow-up advanced imaging, patients with a global brachial plexus palsy had more concentric glenohumeral joints ( Fig. 2 ), while those with upper trunk palsies were more dysplastic ( Fig. 3 ).

**Table 2:** Range of motion

	Elevation			
Variable	Total	Global	Upper trunk	*p* -Value
Preoperative	115.5 ± 40.1	76.7 ± 25.9	144.6 ± 18.2	**< 0.0001**
Postoperative	126.6 ± 40.2	92.2 ± 35.6	150.38 ± 21.8	**0.0002**
*p* -Value	> 0.99	0.333	0.497	
	**External rotation**			
	**Total**	**Global**	**Upper trunk**	***p*** **-Value**
Preoperative	24.8 ± 27.0	32.5 ± 28.7	20.0 ± 24.7	0.3274
Postoperative	36.7 ± 23.4	55.0 ± 14.1	25.4 ± 20.7	**0.003**
*p* -Value	0.144	0.084	0.568	
	**Upper trunk surgical success elevation**			
		**Successful**	**Failed**	***p*** **-Value**
Preoperative		141.4 ± 15.5	149 ± 20.6	0.522
Postoperative		148.8 ± 22.7	153 ± 19.9	0.758
*p* -Value		0.516	0.787	
	**Upper trunk surgical success external rotation**			
		**Successful**	**Failed**	***p*** **-Value**
Preoperative		28.8 ± 24.6	6 ± 17.4	0.125
Postoperative		39.4 ± 11.8	3 ± 8.7	**0.0002**
*p* -Value		0.321	0.766	

Note: Data are shown as mean ± standard deviation.

**Fig. 4:**
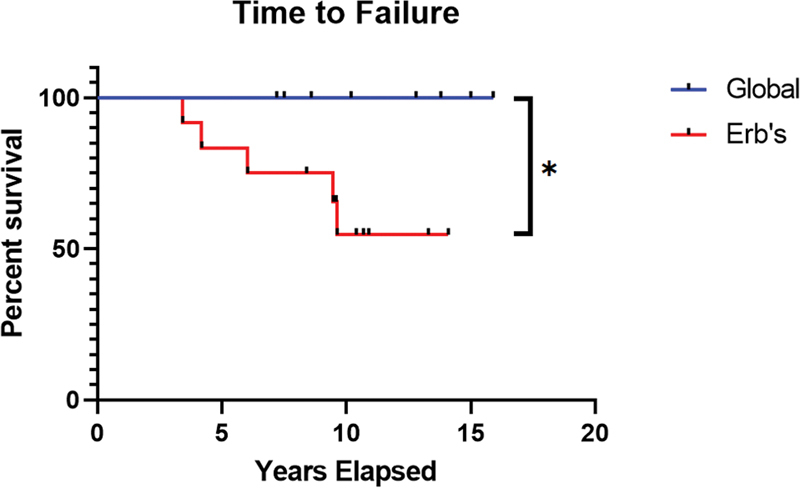
Time to failure.

A Kaplan–Meier estimator for patients that failed surgery demonstrated that all 9 global palsies maintained adequate external rotation without clarion sign at time of final follow-up while 5 of the 13 upper trunk palsies failed isolated latissimus dorsi transfer, which was deemed to be statistically significant ( *p* = 0.0403) ( Fig. 4 ). In these 5 failures, the period from initial transfer to failure averaged 6.6 years, ranging from 3.4 to 9.5 years. All failures were noted to have a positive clarion sign. After failure of the isolated latissimus dorsi transfer, these patients were offered teres major transfer, rotational osteotomy, or by patient's preference, acceptance of their functional status. Three patients accepted their result while two patients chose to undergo a rotational osteotomy.

## Discussion

The present study demonstrated the long-term outcomes possible with isolated latissimus dorsi transfer to the rotator cuff in patients with BPBI, with an average of 11 years' follow-up. All patients with global palsy met the criteria for surgical success at final follow-up while 38% of upper trunk palsy patients met the criteria for failure at an average of 6.6 years from time of surgery ( *p* = 0.0403). Despite this failure, most of the patients chose to accept their final result and deferred revision surgery to assist with their contractures. Improved external rotation at final outcome was the most significant finding associated with surgical success.

There is a paucity of literature on the use of an *isolated* latissimus dorsi transfer for BPBI in children, with a majority of studies reporting short-term results. Most studies with follow-up of 3 years or less demonstrate significant gains in shoulder range of motion and function. van Kooten et al report retrospectively on nine patients with BPBI who underwent latissimus dorsi transfers to restore external rotation of the affected upper extremity, with a mean follow-up of 13.8 months. Seven patients were diagnosed with C5-C6 palsies and two patients were diagnosed with C5-C6-C7 palsies. They found a mean gain of passive external rotation of 12.5 degrees and a mean gain of 28.3 degrees of active external rotation along with a mean gain of active abduction of 22.2 degrees. 17 Similar findings of improved shoulder external rotation and abduction were reported in patients with upper trunk palsy that underwent latissimus dorsi transfer, with a mean follow-up of less than 3 years. 18 19 The results of these initial short-term studies were encouraging, however, follow-up studies have revealed that not all patients maintain these improvements.

Al-Qattan reported on 12 patients with obstetrical brachial plexus injury who underwent latissimus dorsi transfers for external rotation deficits with a mean follow-up of 4 years. In this study, a majority of patients had improved shoulder range of motion with a Mallet score of 4 at final follow-up. However, 2 of the 12 children were noted to have recurrence of loss of active external rotation and decreased Mallet scores of 2. Unfortunately, this study did not identify patients with the type of obstetric brachial plexus injury, that being upper trunk or total. 20 Pagnotta et al then extended observations out to 6 years in a retrospective review of latissimus dorsi transfers in 203 patients including those with C5-C6, C5-C6-C7, and complete birth palsies. Their study did not enumerate the quality of each external rotator and they performed the deltoid splitting approach in the early cases, as well as adding teres major tendons to “pale and thin” latissimus dorsi muscles. Interestingly, they determined that children with C5-C6 palsies had more gains in abduction and external rotation compared with children with C5-C6-C7 and complete palsies. They also found that there was progressive deterioration of abduction at 6 years postoperatively even with preserved active external rotation. 12 Another long-term study in patients undergoing latissimus transfer for obstetric brachial plexus injury similarly found that the initial improvements in range of motion deteriorated over time, at an average of 7.6 years. In this study of 45 patients, 22% required revision surgery. 21 These studies demonstrate that short-term improvements in range of motion with the latissimus transfer may not hold over time, with shoulder abduction and external rotation affected. They also do little to shed light on how outcomes in patients with upper trunk palsy differ from those with global palsy. In this study the outcomes demonstrated improved surgical success for patients with global palsy relative to upper trunk palsy. One possible explanation for this is that in global palsy the internal rotators of the shoulder are more significantly affected and therefore do not contribute to internal rotation contractures as much as is seen in upper trunk palsy.

The current study, which to our knowledge has the longest published average follow-up, agrees with many of the findings of the prior long-term studies. The mean shoulder elevation in all patient groups was not significantly different postoperatively from the preoperative baseline, indicating that the patients' initial improvements deteriorated over time. There was a trend toward preserved shoulder external rotation, as seen in prior studies. This study benefited from a subanalysis of upper trunk versus global palsy and interestingly found that patients with global palsy had better outcomes in the long term with respect to surgical failure. One possible reason for this is that those with global palsies on average had more concentric glenohumeral joints which may be protective against failure compared with upper trunk brachial plexus palsies. Another possible explanation is that shoulder balance after transfer can more easily be obtained in global palsy patients than upper trunk palsy patients due to weaker internal rotator power. Conversely, our study observed the ultimate inadequacy of the latissimus dorsi for external rotation and not for shoulder elevation. The previously quoted studies suggest that with growth in size of these patients, the strength of the latissimus dorsi becomes insufficient. Whether the latissimus dorsi is adequate in strength to maintain external rotation or abduction power has yet to be determined. Thus, we recommend performing isolated latissimus dorsi transfers in global palsy patients only. The question remains if there is an advantage to leaving the teres major in its original anatomic location without the latissimus dorsi.

The limitations of this study include that fact that this is a single-center, single-surgeon study, therefore referral bias could play a role in patient characteristics. Although this study had significant long-term follow-up, the total cohort size was limited and there was no true control group. Additionally, there was limited follow-up imaging to confirm tendon transfer integrity, which was secondary to the desire to limit patient radiation exposure and need for sedation for prolonged imaging.

## Conclusion

The results of this study indicate that patient with global palsy have sustained long-term improved outcomes with isolated latissimus dorsi transfer while patients with upper trunk palsy have a high rate of failure and return to baseline function. Based on these results, we recommend isolated latissimus dorsi transfer for global palsy patients who have isolated infraspinatus weakness. However, given the long-term unpredictable and high failure rate in patients with upper trunk palsy, they may require the use of simultaneous latissimus dorsi and teres major transfers.
